# Heavy vehicle traffic is related to wheeze among schoolchildren: a population-based study in an area with low traffic flows

**DOI:** 10.1186/1476-069X-10-91

**Published:** 2011-10-13

**Authors:** Martin Andersson, Lars Modig, Linnea Hedman, Bertil Forsberg, Eva Rönmark

**Affiliations:** 1 Department of Public Health and Clinical Medicine, Occupational and Environmental Medicine, Umeå University, S-90187 Umeå, Sweden; 2 The OLIN studies, Sunderby Hospital, Luleå, S-97189 Luleå, Sweden

## Abstract

**Background:**

An association between traffic air pollution and respiratory symptoms among children has been reported. However, the effects of traffic air pollution on asthma and wheeze have been very sparsely studied in areas with low traffic intensity in cold climate with poor dispersion. We evaluated the impact of vehicle traffic on childhood asthma and wheeze by objective exposure assessment.

**Methods:**

As a part of the Obstructive Lung Disease in Northern Sweden (OLIN) studies, a questionnaire was sent to the families of all children attending first or second grade in Luleå (72,000 inhabitants) in Northern Sweden in 2006. The age of the children was 7-8 years and the participation rate was 98% (n = 1357). Skin prick tests were performed in 1224 (89%) children. The home addresses were given geographical coordinates and traffic counts were obtained from the local traffic authorities. A proximity model of average daily traffic and average daily heavy vehicle traffic within 200 meters from each participant's home address was used. The associations between traffic exposure and asthma and wheeze, respectively, were analysed in an adjusted multiple logistic regression model.

**Results:**

Exposure to high traffic flows was uncommon in the study area; only 15% of the children lived within 200 meters from a road with a traffic flow of ≥8000 vehicles per day. Living closer than 200 meters from a road with ≥500 heavy vehicles daily was associated with current wheeze, odds ratio 1.7 (confidence interval 1.0-2.7). A dose-response relation was indicated. An increased risk of asthma was also seen, however not significant, odds ratio 1.5 (confidence interval 0.8-2.9). Stratified analyses revealed that the effect of traffic exposure was restricted to the non-sensitized phenotype of asthma and wheeze. The agreement between self-reported traffic exposure and objective measurements of exposure was moderate.

**Conclusions:**

This study showed that already at low levels of exposure, vehicle traffic is related to an increased risk of wheeze among children. Thus, the global burden of traffic air pollution may be underestimated.

## Background

Asthma is a major public health issue, affecting 300 million people all ages world-wide [1]. The global trends in prevalence of asthma among children and young adults describe a diverging pattern, with an on-going increase in developing countries while a prevalence plateau may have been reached in the Western world and high prevalence countries [2-4].

There is a growing body of evidence for vehicle-related air pollution as a determinant of respiratory illness among children. A 2005 WHO-report suggests transport-related air pollution as a risk factor for non-allergic respiratory symptoms [5] and recent cohort studies support this association while indicating an effect of vehicle exhaust on sensitization as well [6-8]. Heavy vehicle traffic has been associated with increased respiratory symptoms among children [9]. However, most of these studies have been performed in areas with high traffic intensity [6,9,10], while results from areas with relatively low traffic intensity and low levels of background air pollution are lacking. A recent report concludes however that evidence of a causal relationship between traffic air pollution and respiratory disease still are insufficient [11].

Exposure assessment is a crucial part of epidemiological studies of respiratory symptoms and morbidity. Self-reported levels of traffic intensity have often been used as a measure of exposure in studies of respiratory symptoms among children. Over-reporting of air pollution exposure by parents of symptomatic children has in some studies been suggested as a source of bias [12,13], but not in others [14].

Within the Obstructive Lung Disease in Northern Sweden studies (OLIN), studies of prevalence, incidence and risk factors for asthma and allergic sensitization among children are in progress since 1996 [15-17]. Recent results include a major increase in allergic sensitization and a moderate increase in physician-diagnosed asthma among children 7 to 8 years of age, from 5.7% in 1996 to 7.4% in 2006, while the prevalence of current wheeze did not change significantly [16]. Self-reporting of living close to heavy trafficked roads was a risk factor for allergic sensitization in 2006, OR 1.3 (CI 1.0-1.6) [15].

The aim of the present study was to investigate the impact of exposure to vehicle traffic outside the home on asthma, wheeze and allergic sensitization among 7-8 years old children in Northern Sweden. A further aim was to evaluate the validity of self-reporting of traffic intensity in comparison with objective exposure assessment.

## Methods

### Study area

Norrbotten, the northernmost part of Sweden, has a relatively dry and cold climate, free from mite and cockroach [15]. The winter lasts from November to March with an average temperature in January of -9 to -17 degrees Celsius. The winter climate causes frequent inversions and thus poor dispersion conditions. In winter-time, the cars are refitted with studded tires. Luleå, with 72,000 inhabitants at the time of the study, is situated by the coast and is the regional capital. The yearly average urban background concentration of NO_2 _is low [18]; 9,7 μg/m^3 ^was reported in 2007 (personal communication with the local environmental authorities). The yearly average street levels of PM_10 _was 16,4 μg/m^3 ^2007, however daily averages above 100 μg/m^3 ^was reported during March and April.

### Study population

A questionnaire was sent to the families of all schoolchildren attending first and second grade, aged 7-8 years, in the municipalities of Luleå, Piteå and Kiruna in 2006 [15,16]. The number of participants was 2585 (96% of invited). In the present study, the children from Luleå (n = 1357, 98% of invited) were further examined. The children in Kiruna and Piteå were not included in the study due to a lack of detailed information on traffic flows. The present study was approved by the Ethics Committee at Umeå University, Sweden.

### Study methodology

The questionnaire consisted of The International Study of Asthma and Allergies in Childhood (ISAAC) core questions [19] extended with questions about physician-diagnoses and symptoms of asthma and allergic diseases, heredity, environment and use of asthma medication [15]. The questionnaire has been validated in 1997 showing a specificity of ≥99% and a sensitivity of 70% regarding the question of physician-diagnosed asthma [20] and very high agreement between parental and teenagers response to questions about asthma and environmental factors [21]. Questions used in the current paper are attached in an appendix.

Skin prick tests (SPT) for ten airborne allergens were performed [15,20], and in Luleå 1224 (89% of invited) were tested. The procedure followed European Academy of Allergology and Clinical Immunology (EAACI) recommendations [22] and has been validated against specific serum-IgE [15]. A mean wheal diameter ≥3 mm was regarded positive. The methodology has been described previously [15]. Allergic sensitization was defined as any positive reaction.

### Objective exposure assessment

The home addresses as well as the schools of the children were assigned to coordinates in a geographical information system (GIS). Data on roads with daily counts of total traffic, as well as heavy traffic, were obtained from the local traffic authorities and later implemented in the GIS-system. Traffic counts were available for all major roads in the study area and also for smaller roads in the city centre, while for a large proportion of the smaller roads within residential blocks traffic counts were missing. With few exceptions the data was collected in 2006 ± 2 years. An exposure model of minimum number of total daily traffic (≥4000, ≥8000), and heavy vehicle traffic (≥100, ≥250, ≥500) on any road within 200 meters from the home addresses was used. For example, " ≥100" means there is at least one road with more than 100 heavy vehicles daily within 200 m from the home address. Children who did not live within 200 m from counted traffic flow were considered unexposed with zero traffic flow.

### Statistical analyses

Odds ratios (OR) with 95% confidence intervals (CI) were calculated by using logistic regression in the unadjusted analysis, and by using stepwise logistic regression when adjusting for possible confounding variables. The PASW Statistics software, v.18.0 (Chicago, IL, USA) was used in all analyses. In the adjusted analysis parental smoking, parental asthma, home dampness, sex and socio-economic status were included into the model, but only variables significantly associated with the outcome or variables that changed the regression coefficient of the exposure variable more than 10% if removed, remained in the final analyses. In the stratified analyses, the results were adjusted for parental asthma. Objective traffic data and self-reported exposure were cross-tabulated to calculate sensitivity and specificity of the question on self-reported exposure.

## Results

### Prevalence of symptoms and exposure

The prevalence of physician-diagnosed asthma was 5.7%, current wheeze 11.2%, and allergic sensitization 26.9%. Among the study population, 22.9% had a road with ≥4000 vehicles per day within 200 meters from the home address and 11.7% had a road with ≥500 heavy vehicles per day within the same radius (Table 1). Of the children, 25.5% were living in an apartment and living in an apartment was significantly associated with higher traffic exposure.

**Table 1:** Prevalence (%) of conditions, risk factors and exposure to vehicle traffic.

		Prevalence % (n)
*Conditions*	Current wheeze	11.2 (152)

	Ever wheeze	22.2 (301)

	Physician-diagnosed asthma	5.7 (78)

	Allergic rhinitis	14.8 (201)

	Allergic sensitization	26.9 (365)

	Allergic sensitization to any pollen	16.7 (227)

		

*Risk factors*	Parental asthma	22.5 (305)

	Parental smoking	21.4 (291)

	Home dampness	12.2 (166)

*Traffic exposure*	≥500 heavy vehicles^a^	11.7 (159)

	≥250 heavy vehicles^a^	20.8 (282)

	≥100 heavy vehicles^a^	31.5 (428)

		

	≥4000 vehicles^b^	22.9 (311)

	≥8000 vehicles^b^	15.1 (205)

		

	Self-reported exposure^c^	30.1 (408)

^a ^Number of heavy vehicles daily within 200 meters from the home (Heavy traffic)

^b ^Total number of vehicles daily within 200 meters from the home (Total traffic)

^c ^A large busy road or a very frequented bus stop within 200 meters of the home

### Current wheeze

**Figure 1:**
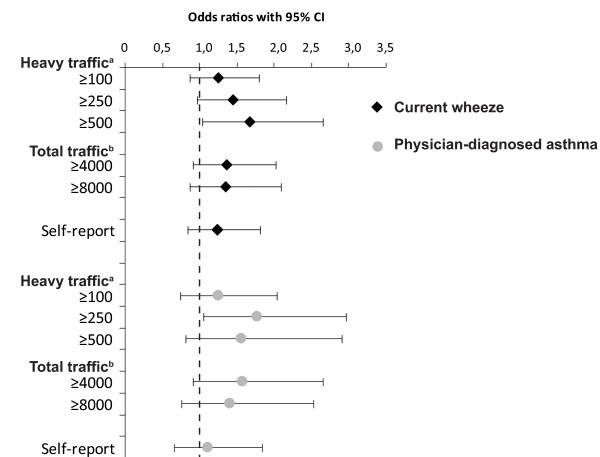
**Traffic exposure as a risk factor for current wheeze and physician-diagnosed asthma respectively**. Risk expressed as adjusted odds ratios (OR 95%CI), calculated by multiple logistic regression analyses and adjusted for parental smoking, parental asthma, home dampness, sex and socio-economic status.^a ^Number of heavy vehicles daily within 200 meters from the home^b ^Total number of vehicles daily within 200 meters from the home.

The unadjusted odds ratio for current wheeze when living closer than 200 m from a road with ≥500 heavy vehicles daily was 1.8 (95% CI 1.2-2.9). This association became weaker but still significant, with decreasing frequency of heavy traffic flow. A significant association between total traffic flow and current wheeze was also found, OR 1.5-1.6 (Table 2). The risk pattern was verified by the adjusted analysis with an OR of 1.7 (CI 1.0-2.7) for those living within 200 m from ≥500 heavy vehicles daily and OR 1.5 (CI 1.0-2.2) for ≥250 heavy vehicles. Similarly total traffic flow yielded ORs of 1.4 and borderline significant (Figure 1). No associations between traffic exposure at school and wheeze or asthma were found.

**Table 2:** Traffic exposure as a risk factor for wheeze, asthma, allergic rhinitis and allergic sensitization expressed as unadjusted odds ratio (OR 95%CI).

Traffic exposure		Current wheeze	Ever wheeze	Physician-diagnosed asthma	Allergic rhinitis	Allergic sensitization	Allergic sensitization to any pollen
Heavy traffic^a^	≥100	1.38 (0.97-1.95)	1.22 (0.93-1.60)	1.29 (0.80-2.08)	1.25 (0.91-1.71)	1.13 (0.87-1.47)	0.98 (0.72-1.33)

	≥250	1.52 (1.04-2.23)	1.18 (0.87-1.60)	1.74 (1.06-2.87)	1.23 (0.87-1.76)	1.35 (1.00-1.81)	1.23 (0.87-1.73)

	≥500	1.84 (1.17-2.88)	1.30 (0.89-1.90)	1.70 (0.93-3.10)	0.97 (0.60-1.55)	1.12 (0.77-1.63)	1.22 (0.79-1.88)

Total traffic^b^	≥4000	1.58 (1.09-2.29)	1.21 (0.90-1.63)	1.62 (0.99-2.66)	1.36 (0.97-1.91)	1.19 (0.90-1.59)	1.10 (0.78-1.54)

	≥8000	1.51 (0.98-2.30)	1.09 (0.76-1.55)	1.48 (0.83-2.61)	1.07 (0.71-1.62)	1.09 (0.77-1.53)	1.10 (0.74-1.64)

Self-reported^c^		1.39 (0.98-1.99)	1.52 (1.16-1.99)	1.31 (0.81-2.11)	1.52 (1.11-2.09)	1.38 (1.06-1.80)	1.25 (0.92-1.70)

^a ^Number of heavy vehicles daily within 200 meters from the home (Heavy traffic)

^b ^Total number of vehicles daily within 200 meters from the home (Total traffic)

^c ^A large busy road or a very frequented bus stop within 200 meters of the home

No significant associations of daily traffic counts and ever wheeze was found (Table 2). Since exposure to ≥250 heavy vehicles was significantly associated with allergic sensitization, the analyses were additionally adjusted for allergic sensitisation. These results remained similar and current wheeze was associated with living within 200 m from ≥250 heavy vehicles, OR 1.2 (CI 0.8-1.8), and ≥500 heavy vehicles, OR 1.7 (CI 1.1-2.8).

**Figure 2:**
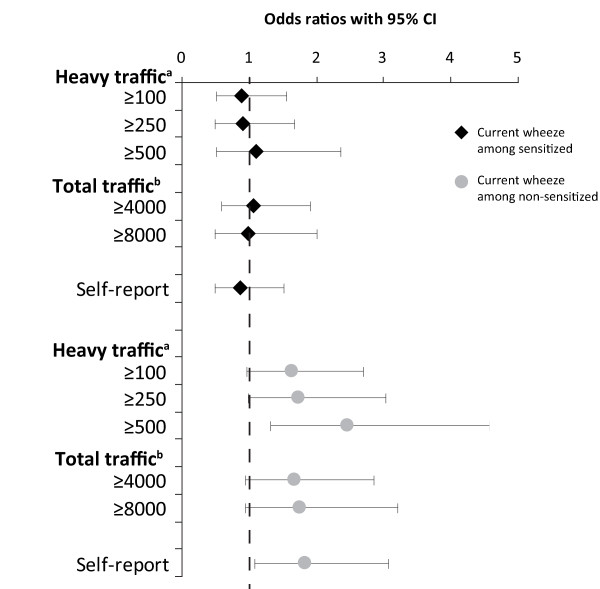
**Traffic exposure as a risk factor for current wheeze, stratified by allergic sensitization**. Risk expressed as adjusted odds ratios (OR 95%CI), calculated by multiple logistic regression analyses and adjusted for parental asthma.^a ^Number of heavy vehicles daily within 200 meters from the home^b ^Total number of vehicles daily within 200 meters from the home.

Stratified analyses among sensitized and non-sensitized children, respectively, showed that traffic exposure was a significant or border-line significant risk factor for current wheeze only among non-sensitized children, with OR ranging from 1.6 to 2.4 depending on which exposure variable in use (Figure 2).

### Physician-diagnosed asthma

**Figure 3:**
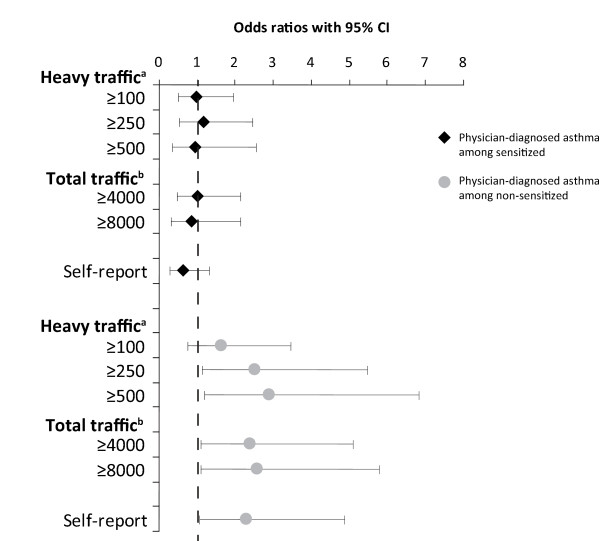
**Traffic exposure as a risk factor for physician-diagnosed asthma, stratified by allergic sensitization**. Risk expressed as adjusted odds ratios (OR 95%CI), calculated by multiple logistic regression analyses and adjusted for parental asthma.^a ^Number of heavy vehicles daily within 200 meters from the home^b ^Total number of vehicles daily within 200 meters from the home.

Living close to heavy traffic was associated with physician-diagnosed asthma, however significantly so only in the analysis of the ≥250 heavy vehicles group, unadjusted OR 1.7 (CI 1.1-2.9) and adjusted OR 1.8 (CI 1.1-3.0). Among those children living closer than 200 m from ≥500 heavy vehicles daily, the unadjusted OR was 1.7 (CI 0.9-3.1), and the adjusted OR was 1.5 (CI 0.8-2.9). As for current wheeze, significant associations between exposure from traffic and physician-diagnosed asthma were only found among non-sensitized children with OR between 2.3 to 2.9 (Figure 3).

### Allergic sensitization

Allergic sensitization was significantly associated with living closer than 200 m from ≥250 heavy vehicles, OR 1.4 (CI 1.0-1.8), however no dose-response relationship was found. No significant association between unspecified traffic flows and allergic rhinitis, sensitization to at least one allergen, or sensitization to any pollen was found (Table 2). Traffic exposure measured objectively was not associated with eczema while self-reported exposure was, OR 1.3 (CI 1.0-1.7).

A sensitivity analysis using 100 meters instead of 200 meters yielded similar results, however less powered.

### Self-reported exposure

The sensitivity of "Self-reported exposure" varied between 50.1% and 66.9% and the specificity ranged from 73.5% to 77.5% depending on which exposure-level used as the "true exposure". The sensitivity increased and the specificity decreased with increasing traffic flows both regarding heavy vehicle and total vehicle flow. There were significant associations of self-reported exposure and ever wheeze, allergic rhinitis and allergic sensitization (Table 2).

## Discussion

We found significant associations between vehicle traffic flows and wheeze and asthma among schoolchildren. The effect was most pronounced for heavy traffic and a dose-response relationship was indicated regarding wheeze. The associations remained after adjusting for possible confounding factors such as heredity for asthma, parental smoking, home dampness, sex and socio-economic status. The analyses were less powered regarding asthma; however, the results indicated an association between exposure and physician-diagnosed asthma. Furthermore, the effect of traffic exposure was restricted to the non-sensitized phenotypes of asthma and wheeze.

The present study is one of the very few that describe an association between traffic air pollution and symptoms of asthma in children in a cold climate setting with, in a global perspective, low traffic intensity and small socio-economic differences in the population. We found increased risks comparable, or even more pronounced, to studies in far more traffic-intense areas [10,23]. Already at exposures defined as living within 200 meters from a road with ≥250 heavy vehicles per day we found significant associations, and only 15% of the cohort were exposed to more than 8,000 vehicles per day. This can be compared to studies defining exposed participants as those living 50 meters from a highway with ≥30,000 vehicles per day [10], or where the minimum of exposure was 30,000 vehicles per day [9]. However, due to poor dispersion conditions in winter, the gradients in traffic pollution within the city are expected to be strong in our study area.

Several studies, using different exposure classifications, have found an association between childhood respiratory symptoms and residential proximity to major roads [10,14,23-25]. Our results are in line with the suggestion of heavy vehicle traffic, mainly diesel engines, to be an inducer of asthmatic symptoms [23]. In line with other studies [7], the effect of traffic exposure was weaker for asthma compared to wheeze. Transport-related air pollution generates both gases and particles, which after inhalation might cause direct damage or contribute to oxidative stress and airway inflammation. The potential to cause inflammation has been suggested as a possible mechanism for the impact of traffic exhaust on respiratory symptoms and asthma [26]. Children with asthma have been shown to have decreased levels of anti-oxidant defense components [27] and may thus be a sensitive group. Transport-related air pollution consists of many potentially toxic substances and it is often not possible to differentiate these in epidemiological studies. However, diesel exhaust has been suggested as an inducer of airway inflammation [28].

It has been suggested that the expected number of people developing asthma and asthmatic symptoms due to traffic exposure may have been underestimated in studies of highly trafficked areas due to a high level of background pollution. Studies conducted in less polluted areas are therefore important. A study in Jimma, Ethiopia, tried to address this concern, showing an increased risk of wheezing in relation to road proximity in this in general low traffic area [29]. Their findings are in line with our results, however derived from a very different area compared to ours, with regard to climate, socio-economics, as well as the standard and emissions of vehicle engines. In our study area, the yearly average urban background concentration of NO_2 _was low; 9,7 μg/m^3 ^was reported in 2007 by the local environmental authorities (personal communication). However, the use of studded tires to prevent car accidents in the winter implies an additional source of particulate air pollution in this part of Scandinavia. When using a geographical exposure metric the associations to health effects cannot be attributed any specific pollutant other than traffic pollution as a whole. The effects seen could therefore be due to long-term exposure to particles or gases, or even to high levels of coarse particles during short episodes in the spring. Children having high traffic flows within 200 meters from home, are most often exposed to higher levels of gases and particles, both refereeing to long- and short term exposures.

Low socio-economic status has been associated with asthma in childhood [30], and may be related to area of residence [10]. As we lacked information about parental education or income, living in an apartment in contrast to living in a single family house was used as a proxy variable for low socioeconomic status. In Sweden, the majority of those living in apartment buildings belong to the lowest income quartile [31]. In Luleå, rental apartments were more common than condominiums, and at the time of the study there were very few expensive condominiums that required a high income. SES was related to traffic exposure in our study as in others [32], we have corrected the analyses accordingly. However, unmeasured socioeconomic confounding may still exist as well as other confounding factors. Although the area has a well-developed district heating system, the use of wood stoves is not uncommon, especially in the countryside and in winter-time. We are however lacking the data needed to properly assess this potential confounder.

Allergic sensitization was associated with living within 200 m from a road with more than 250 heavy vehicles daily, while no dose-response relationship was found. An association between traffic exhaust and allergic sensitization has been suggested, but the results are inconsistent [25]. However, our stratified analyses of asthma and wheeze among sensitized and non-sensitized children, respectively, revealed that traffic exposure was only related to asthma and current wheeze among non-sensitized children (Figure 2 and 3). This result is in line with other studies [33] as well as a previous OLIN study among children in the same areas and the same ages. Environmental risk factors, such as house dampness and mothers' smoking, were not associated with allergic asthma, but only with non-allergic asthma [20]. The reason for the discrepancy could be explained by the fact that asthma is a syndrome with several phenotypes and not a homogenous disease. As allergic sensitization is the most important risk factor for asthma, other factors such as environmental exposures will thus be less important among sensitized subjects. In the Netherlands, respiratory symptoms were associated with children going to schools located near motorways with high traffic counts of heavy vehicles. In contrast to our findings, the adverse health effect was mainly restricted to allergic, sensitized or bronchial-hyperreactive children [9]. In our study, traffic exposure at school was not related to asthma or wheeze probably due to short exposure time at school. At 7-8 years of age children spend few hours per day at school and they had only attended school for 0.5-1.5 years when the study was performed.

The analyses of self-reported traffic exposure showed significant associations with ever wheeze, allergic rhinitis and allergic sensitization. As discussed by others, such associations could be a result of reporting bias where symptomatic subjects are more aware about the environment [12,13]. On the other hand our validation analysis of the question on self-reported traffic exposure showed an acceptable specificity while the sensitivity was lower. Thus the effect of traffic exposure based on self-reported data probably was underestimated.

The strength of our study is mainly the objective exposure assessment together with the use of a validated questionnaire regarding symptoms and diagnoses. Further, and importantly, the participation rate was very high, which practically eliminates the risk of selection bias. The exposure assessment was based on objective measurements of traffic flows together with GIS-derived distances to home addresses, and information on traffic counts on almost every larger street was available. The availability of traffic counts provide a high spatial resolution of exposure as opposed to relying on data from a few monitoring stations, and therefore reduces misclassification of exposure. Objective exposure assessments also limit the impact of report bias, which has been suggested as an important source of bias in studies of road traffic and children's respiratory health [34]. Further, similar GIS exposure models are well established in studies of traffic related air pollution and respiratory health. There are alternative methods for describing exposure with high spatial resolution, where Land Use Regression (LUR) and meteorological dispersion models are most common. Traffic is the main emission source of air pollution in Swedish cities, and the most influencing parameter in both LUR and dispersion models. There is yet no established dispersion model or LUR-model for this area, which otherwise could have been used as an interesting complement to the geographical exposure metric.

A longitudinal study design and information about residential history would further strengthen the results regarding the association causality as a cross-sectional study limit the causal interpretation. The questionnaire-based study design without objective assessment of symptoms and diagnoses, except the skin prick tests, could be regarded as a limitation. However, the ISAAC core questions have been used extensively in international studies of childhood asthma, and a validation study for self-reported physician-diagnosed asthma versus paediatricians assessment of asthma has previously been carried out, showing very high specificity and 70% sensitivity for the question about physician-diagnosed asthma [20]. Further, the prevalence of asthma and wheeze is in line with reports from other studies of similar ages in Sweden and other Northern European countries [3,35].

In our exposure variables, we used different cut-off levels of daily total and heavy vehicle traffic counts, respectively, on any road within a fixed radius of 200 m. To compare children living at different distances from a highly trafficked road was not possible due to the limited number of children living within 50 or 100 m from such roads in our study area. The main draw back with this approach refers to the knowledge of vehicle exhaust declining rapidly with increasing distance from the road. However, we assumed that high traffic flow within 200 meters from home still was close enough to contribute to the children's exposure in this flat coastal area. The best size of the radius can of course always be discussed, and most likely it should be different in different cities depending on building structure and building density. Choosing a smaller radius reduced the amount of children exposed and consequently also the statistical power. The sensitivity analysis using a smaller radius showed similar results but they did not reach statistical significance. By using three different cut-off levels within the 200 m radius we could show the importance of larger roads (≥250 vehicles per day) in comparison to using a lower cut-off (≥100 vehicles per day).

## Conclusions

We have found an association between vehicle traffic flow and wheeze among schoolchildren in a city in Northern Sweden, where the over-all traffic intensity is low. The results indicate that vehicle traffic emissions may pose a threat to public health also in large areas of the world where background pollution and traffic intensity are low and an even larger threat to respiratory health in highly trafficked communities where the risk may be underestimated.

## List of Abbreviations

EAACI: European Academy of Allergology and Clinical Immunology; GIS: Geographical Information System; ISAAC: The International Study of Asthma and Allergies in Childhood; m: meters; OLIN: Obstructive Lung Disease in Northern Sweden Studies; OR: Odds ratio; SPT: Skin Prick Test

## Competing interests

The authors declare that they have no competing interests.

## Appendix

The definitions was based on the following questions:

Current wheeze: "Has your child had wheezing or whistling in the chest in the last 12 months?" [18]

Ever wheeze: "Has you child ever had wheezing or whistling in the chest?" [18]

Physician-diagnosed asthma: "Has your child been diagnosed by a physician as having asthma?" [14]

Allergic rhinitis: "Has the child during the last 12 months had sneezing, runny nose or nasal obstruction without having had a common cold?" [18]

Eczema: "Has your child during the last 12 months had an itchy rash that was coming and going for at least six months?

Parental smoking: Father and/or mother smokes [14].

Parental asthma: Father and/or mother with asthma [14].

Home dampness: Previously or currently home dampness [14].

Socio-economic status (SES): Living in a single family house versus in an apartment [14].

Self-reported traffic exposure: A large busy road or a very frequented bus stop within 200 meters of the home [14].

## Authors' contributions

MA participated in the design, statistical analysis and interpretation of the results, and drafted the manuscript. LM participated in the design and critically revised the manuscript. LH participated in the acquisition of data and critically revised the manuscript. BF participated in the design and critically revised the manuscript. ER designed the study, participated in acquisition of data, statistical analysis, and interpretation of the results and helped to draft the manuscript. All authors have read and approved the final manuscript.
